# Urokinase-derived peptide UP-7 suppresses tumor angiogenesis and metastasis through inhibition of FAK activation

**DOI:** 10.18632/oncotarget.24131

**Published:** 2018-01-10

**Authors:** Hyun-Kyung Kim, Purevjargal Naidansuren, Seung Woo Lee, Rae-Kwon Kim, Su-Jae Lee, Suk Keun Lee, Yong-Kil Hong, Young Ae Joe

**Affiliations:** ^1^ Cancer Research Institute, College of Medicine, The Catholic University of Korea, Seoul, 06519, Republic of Korea; ^2^ Department of Medical Lifescience, College of Medicine, The Catholic University of Korea, Seoul, 06519, Republic of Korea; ^3^ Cancer Evolution Research Center, College of Medicine, The Catholic University of Korea, Seoul, 06519, Republic of Korea; ^4^ Department of Life Science, Research Institute for Natural Sciences, Hanyang University, Seoul, 04763, Republic of Korea; ^5^ Department of Oral Pathology, College of Dentistry, Kangnung National University, Gangneung, Gangwon-do, 25457, Republic of Korea

**Keywords:** angiogenesis inhibitor, urokinase-type plasminogen activator, peptide, breast cancer, metastasis

## Abstract

The recombinant kringle domain of urokinase (UK1) has been shown to inhibit angiogenesis and brain tumor growth *in vivo*. To avoid limitations in application due to mass production of recombinant protein, we attempted to develop a novel peptide inhibitor from UK1 sequence consisting of 83 amino acids that contains α-helices, loops and β-sheets. We dissected UK1 sequence to seven peptides based on structure and amino acid characteristics, and examined the anti-angiogenic activities for the constructed peptides. Among the tested peptides, UP-7 most potently inhibited the proliferation and migration of endothelial cells (ECs) *in vitro*, and also potently inhibited *in vivo* angiogenesis in the mouse matrigel plug assay. Such anti-angiogenic activities were not exerted by the scrambled peptide. At molecular level, UP-7 inhibited growth factor-induced phosphorylation of FAK and ERK1/2. It also suppressed formation of stress fibers and focal adhesions and also inhibited the attachment and spreading of ECs onto immobilized fibronectin. In a lung cancer animal model xenografted with non-UP-7-sensitive NCI-H460 cells, systemic treatment of UP-7 effectively suppressed tumor growth through inhibition of angiogenesis. Interestingly, breast cancer cells such as LM-MDA-MB-231 cells were moderately sensitive to UP-7 in proliferation differently from other cancer cells. UP-7 also inhibited migration, invasion and FAK phosphorylation of LM-MDA-MB-231 cells. Accordingly, UP-7 potently inhibited lung metastatic growth of LM-MDA-MB-231 cells in an experimental metastasis model. Taken together, these results suggest that novel peptide UP-7 can be effectively used for treatment of breast cancer metastatic growth through inhibition of angiogenesis and invasion.

## INTRODUCTION

Metastasis is the main cause of mortality in most cancer patients including breast cancer patient [1]. It occurs through a multistep process including detachment of primary tumor cells, degradation of basement membranes, migration, invasion, adhesion, and proliferation at secondary site [2]. Tumor cell migration is a key process for metastasis, and the process is regulated through cytoskeletal and cell extracellular matrix (ECM) adhesion remodeling mediated by signal [3, 4].

Focal adhesion kinase (FAK) is a cytoplasmic tyrosine kinase that plays an important role in integrin and growth factor mediated signaling. FAK activation requires integrin receptor clustering by the integrin-mediated binding of cells to ECM proteins. Integrins can integrate cues from the ECM and growth factors to regulate complex biological process including tumor growth, metastasis and angiogenesis [5]. Tumor-associated endothelial cells and several tumor samples including breast cancer display increased FAK mRNA and activated FAK, and FAK promotes tumor progression and metastasis through effects on cancer cells and stromal cells [6]. Endothelial FAK-deletion in adult mice resulted in suppression of tumor growth and angiogenesis [7]. FAK regulates cell migration by controlling the turnover of focal adhesion contacts [8]. Cells recovered from FAK-deficient mice show reduced motility *in vitro* [8], whereas overexpression of FAK increases cell migration on fibronecin (FN) [9]. Fibronectin plays a role in creation of the premetastatic niche before metastasis [10].

Angiogenesis is a critical step in the progression of tumors by supplying nutrients and oxygen, and occurs by multistep processes mediated by integrin-ligated cellular activities [11, 12]. Tumor-associated vessels show increased expression of integrins αvβ3 and αvβ5 that allow angiogenic endothelial cells (ECs) to bind provisional matrix protein such as vitronectin, fibrinogen, von Willebrand factor, osteopontin and FN, thereby providing survival cues and traction for invading cells [13, 14]. Thus, integrins have been challenged as targets for cancer therapy through control of angiogenesis and tumor growth, and several integrin antagonists have been developed as monoclonal antibodies and RGD peptide mimetics such cilgengitide, the αvβ3 and αvβ5-directed antagonist. However, ligand sequence-based RGD mimetics paradoxically stimulate tumor growth and angiogenesis at low concentrations by acting as integrin agonist [15], and cilengitide was not efficacious in clinical trials of patients with glioblastoma [16]. In addition, inherent/acquired resistance to anti-angiogenic agents have been identified in preclinical models [17]. Tumor and tumor microenvironment have been known to be involved in VEGF-independent pathways mediating resistance to VEGF-A inhibitors. Thus, development of a novel anti-angiogenic peptide with various mechanism of inhibitory action is necessary.

Previously, we have shown that the recombinant kringle domain of urokinase (UK1) is a potent angiogenesis inhibitor and it inhibits tumor growth in a brain tumor model [18, 19]. However, potential problems such as poor bioavailability, antigenicity and inconsistency in bioactivity from batch to batch during production of recombinant protein can hamper the application of UK1 to clinical practice [20]. To overcome these disadvantages, we investigated which peptides derived from UK1 have anti-angiogenic activities to develop a novel small size anti-angiogenic peptide. Among UK1 derived 7 peptides, UP-7 that has anti-parallel beta sheet structure showed the most potent anti-proliferative and anti-migratory effects on ECs. In this study, we assessed the effect of UP-7 on anti-angiogenic, anti-tumorigenic, and anti-metastatic activities using *in vitro* and *in vivo* models. We found that UP-7 peptide potently inhibits not only angiogenesis-dependent tumor growth, but also invasion and metastasis of breast cancer through inhibition of FAK activation.

## RESULTS

### Evaluation of the anti-angiogenic activities of UK1-derived peptides

To identify the anti-angiogenic epitope from UK1, the whole sequence of UK1 was dissected into 7 peptides considering its structure and characteristics of amino acid residues. In consideration of three dimensional structure of UK1, amino acid residues exposed to the surface were chosen. UP-1 (^3^Glu-^9^Tyr), UP-5 (^46^Gly–^50^Tyr), and UP-6 (^54^Pro–^60^Arg) sequences are characterized by loops in urokinase kringle structure. UP-3 (^24^Pro–^38^His) and UP-4 (^42^Ser–^48^Gly) sequences contain short helical structure, whereas UP-2 (^14^Ser–^22^Pro) sequence has rudimentary β-sheet structure. UP-7 (^61^Pro–^74^Glu) sequence was characterized as extended antiparallel β-sheet structure [21] (Supplementary Figure 1). To select the most potent anti-angiogenic peptide sequence, the constructed peptides were assessed for their inhibitory effects on proliferation and migration of human umbilical vein endothelial cells (HUVECs) at 1, 10, and 100 µM. All the tested peptides showed dose-dependent effects and the most potent inhibitory effects at 100 µM. When we compared their inhibitory effects at 100 µM, UP-7 displayed the highest inhibitory activities among the tested peptides (Supplementary Table S1). Therefore, we chose the UP-7 for further assessment for anti-angiogenic activity.

### UP-7 peptide potently inhibits angiogenesis *in vitro* and *in vivo*

**Figure 1:**
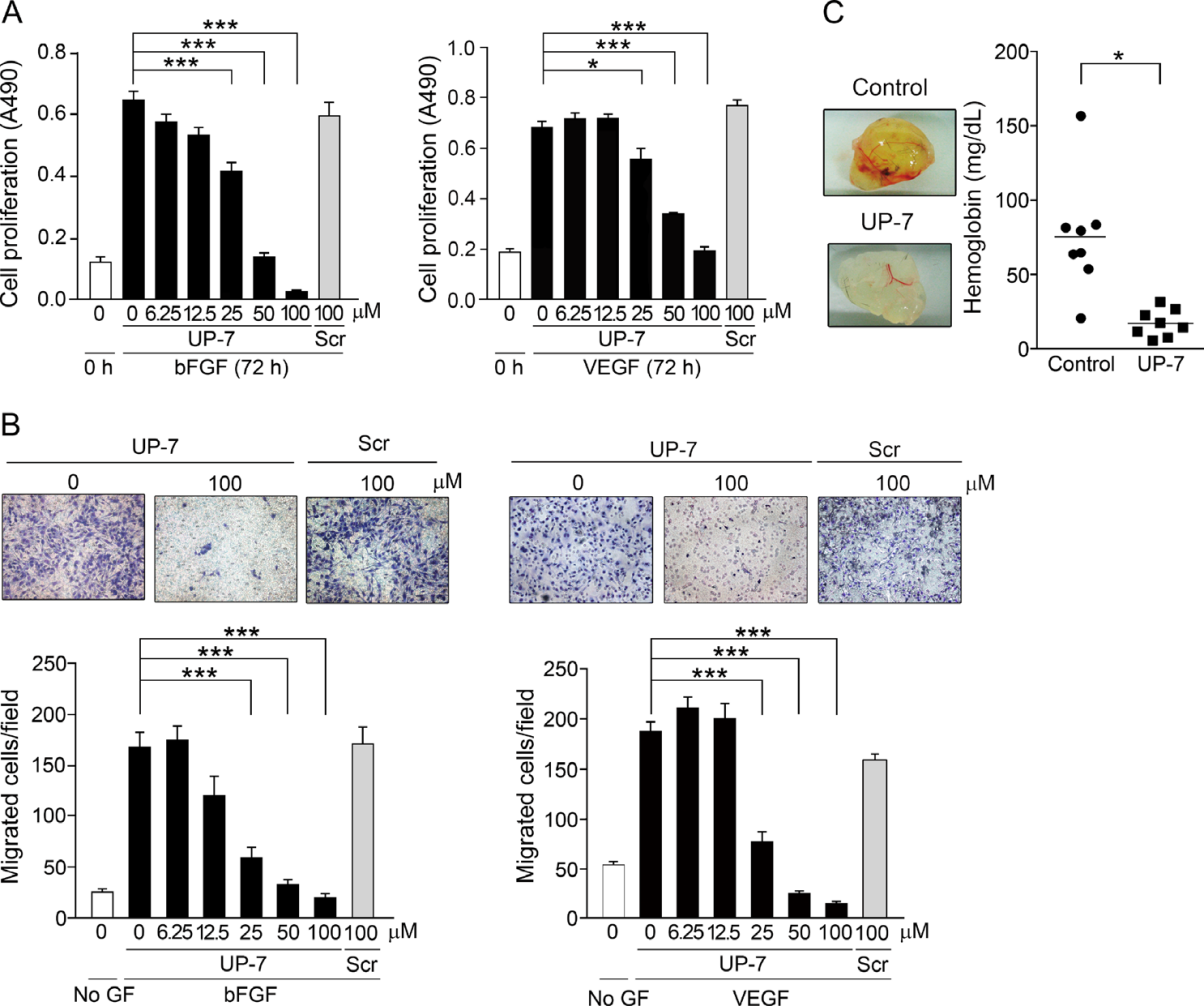
UP-7 potently inhibits angiogenesis *in vitro* and *in vivo* (**A**) Serum-starved HUVECs were treated with the indicated concentrations of UP-7 or Scr, followed by the stimulation with 3 ng/ml bFGF (left) or 5 ng/ml VEGF (right) and 5% FBS. After 72 h, MTS assay was performed. (**B**) Serum-starved HUVECs pretreated with UP-7 or Scr for 30 min was allowed to migrate through Transwell membranes toward bFGF (10 ng/ml) for 8 h or VEGF (5 ng/ml) for 5 h. Representative fields (upper panel) and bar graph (lower panel) of the number of the migrated cells are presented. GF: growth factor. (**C**) Matrigel plug containing bFGF and VEGF together with/without UP-7 was injected into mouse flank. After 7 days, the Matrigel plug was removed and photographed (left panel). Graph indicates the hemoglobin contents within Matrigel plugs (right panel). ^*^p < 0.05; and ^***^p < 0.001.

The efficacy of UP-7 was further evaluated by proliferation and migration assays. As shown in Figure 1A, UP-7 significantly inhibited bFGF- or VEGF- induced proliferation of HUVECs in a dose dependent manner (IC_50_ = 25 µM and IC_50_ = 40 µM, respectively), whereas the scrambled (Scr) (VPQQWKLYCEGVLPV) peptide showed no effect on cell proliferation even at 100 µM. In a modified Boyden chamber assay, treatment of HUVECs with UP-7 significantly inhibited bFGF- or VEGF-induced HUVEC migration dose-dependently (IC_50_ = 16 µM and IC_50_ = 15 µM, respectively), whereas Scr (100 µM) showed no inhibitory effect (Figure 1B). Unexpectedly, UP-7 was not able to inhibit tube formation on Matrigel-coated plates differently from TP-7, the antiparallel β sheet-derived peptide of tissue type-plasminogen activator (Supplementary Figure 2) [22].

Next, to assess whether the UP-7 is able to inhibit angiogenesis *in vivo*, Matrigel plug assay was performed in C57BL/6 mice. Mice were injected with Matrigel containing with VEGF, bFGF, and heparin with or without UP-7 (100 µM). After 7 days, the implanted Matrigel was removed and examined. Untreated control group showed red colored Matrigel plugs and their hemoglobin content was 75.6 ± 13.6 mg/dL (Figure 1C). On the other hand, treatment with UP-7 resulted in non-red plugs and the concentration of hemoglobin was much lower (17.4 ± 3.2 mg/dL), representing a 76.9% reduction compared to control group. Thus, these data indicate that UP-7 has potent anti-angiogenic activities *in vitro* and *in vivo*.

### UP-7 blocks FAK and ERK1/2 phosphorylation and cytoskeletal rearrangement in ECs

**Figure 2:**
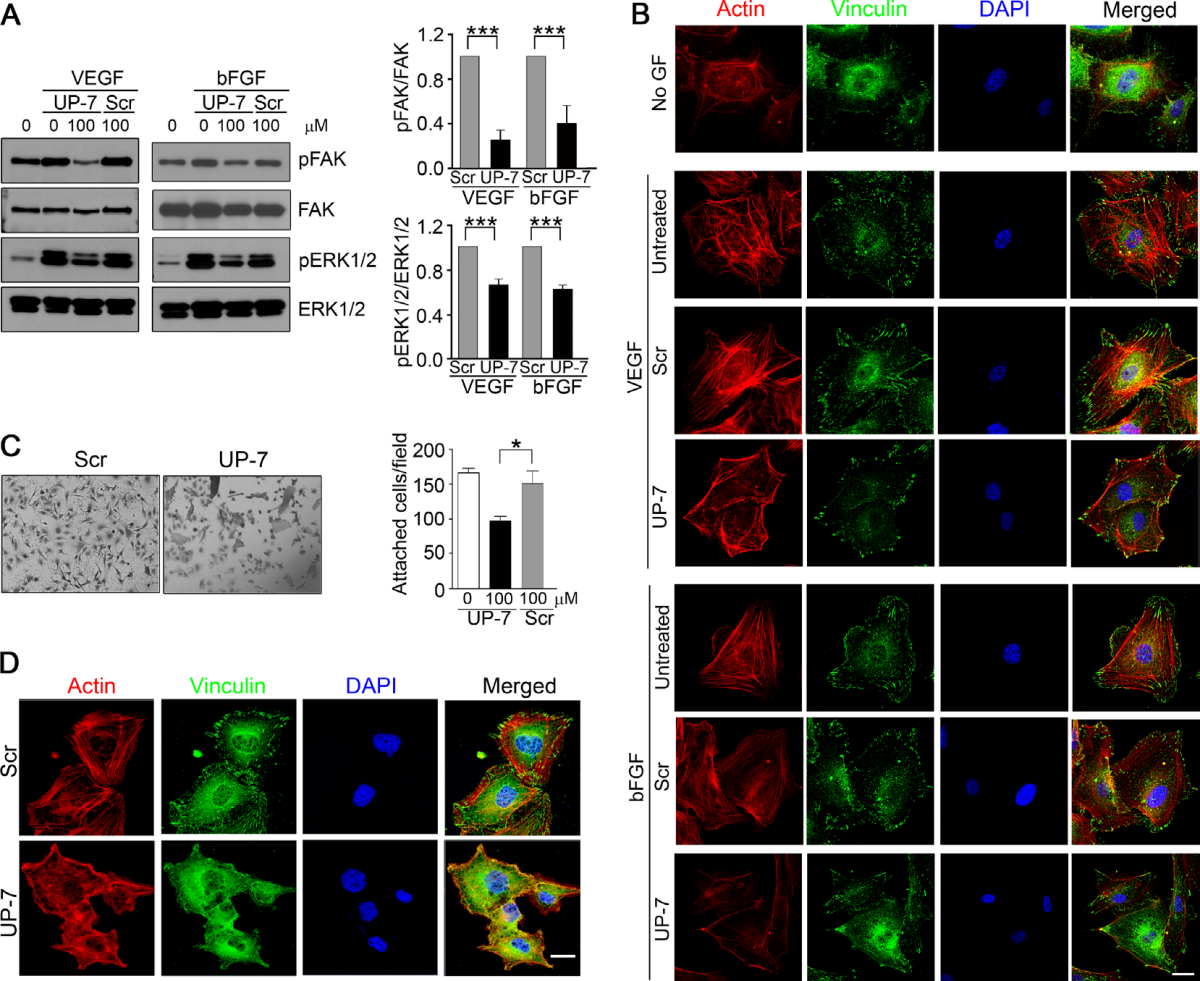
UP-7 inhibits phosphorylation of FAK and ERK1/2 and formation of actin stress fibers and focal adhesion in ECs (**A**) Serum-starved HUVECs were pretreated with UP-7 or Scr (100 µM) for 30 min and stimulated with VEGF (5 ng/ml) or bFGF (3 ng/ml) for 10 min, followed by western blot analysis. Quantitative analysis of three independent experiments is shown. (**B**) HUVECs were pretreated with UP-7 or Scr (100 µM) for 30 min and stimulated with VEGF or bFGF for 10 min. Then, the cells were immunostained with anti-vinculin antibody and Alexa 488 conjugated secondary antibody, followed by staining with TRITC-conjugated phalloidin and DAPI. Scale bar = 20 μm. (**C**, **D**) Serum-starved HUVECs were pretreated with UP-7 or Scr (100 µM) for 30 min and plated on FN (20 μg/ml)-coated plates for 90 min. The attached cells were stained with crystal violet and counted. Representative images (left panel) and the number of attached cells (right panel) are shown (C). The attached cells were immunostained with anti-vinculin antibody and Alexa 488-conjugated secondary antibody, followed by staining with TRITC-conjugated phalloidin and DAPI (D). Scale bar = 20 μm. ^*^p < 0.05; and ^***^p < 0.001.

We previously reported that UK1 inhibits phosphorylation of ERK1/2 by VEGF and bFGF in HUVEC [23, 24]. To test whether UP-7 also affects the phosphorylation of FAK and ERK1/2 in HUVECs, serum-starved HUVECs were pretreated with indicated concentrations of UP-7 for 30 min and then stimulated with either VEGF or bFGF for 10 min. As shown in Figure 2A, UP-7 significantly inhibited phosphorylation of FAK and ERK1/2 induced by both VEGF and bFGF, while Scr had no effects. Since cell migration involves cytoskeleton rearrangement, we tested the effect of UP-7 on cytoskeletal rearrangement. As shown in Figure 2B, VEGF or bFGF treatment induced formation of actin stress fibers in untreated HUVECs and the formed stress fibers were anchored at focal adhesions as shown in the co-localization of actin and vinculin. However, pretreatment with UP-7 before growth factor stimulation disrupted both the formation of actin stress fibers and focal adhesion complexes, whereas Scr showed no inhibitory effects. Thus, these data indicate that UP-7 effectively inhibits activation of FAK and ERK1/2, and cytoskeleton rearrangement induced by both VEGF and bFGF. Endothelial cell adhesion to ECM and appropriate cellular shape by attachment are crucial for endothelial cell growth, differentiation and migration [25, 26]. The early stages of sprouting angiogenesis have been known to progress in a circumstance rich in collagen with fibrin and FN [12]. To test whether UP-7 affects adhesion of HUVECs to an immobilized FN, cell adhesion experiments were performed in the presence of UP-7 or Scr. As shown in Figure 2C, UP-7 (100 µM) significantly inhibited HUVEC adhesion and spreading onto FN and the adhesion was reduced by about 40% compare to untreated control. In contrast, equal concentration of Scr had no effect. In addition, UP-7 treatment disrupted formation of actin stress fibers and focal adhesions upon cellular attachment to FN compared to Scr-treated cells (Figure 2D). In case of collagen I matrix, there was no significant inhibitory effect of UP-7 on cellular attachment, but significant disturbance was observed in formation of focal adhesion complex (Supplementary Figure 3). These findings suggest that UP-7 may inhibit cellular function in part through perturbing interaction of cells with ECM.

### UP-7 inhibits proliferation of breast cancer cells, but not other cancer cells

**Figure 3:**
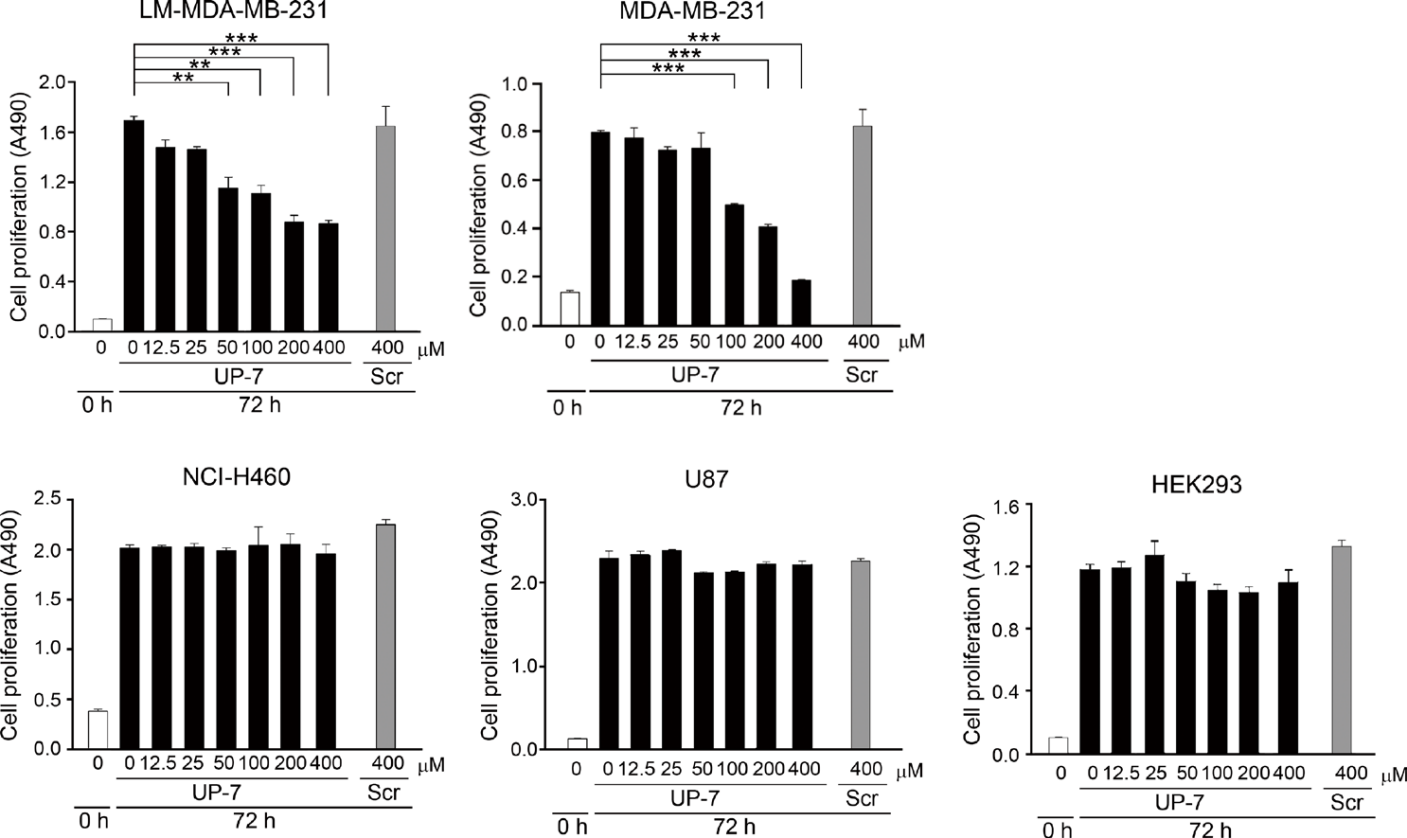
The differential effects of UP-7 on cancer cells The effect of UP-7 on proliferation of several cancer cells was performed using MTS assay. LM-MDA-MB-231, MDA-MB-231, NCI-H460, U87 or HEK293 cells were treated with the indicated concentrations of UP-7 or Scr and cultured for 72 h, followed by MTS assay. ^**^p < 0.01; and ^***^p < 0.001.

Next, it was questioned whether UP-7 could also affect proliferation of cancer cells besides anti-endothelial cell activity. To do this, several cancer cells were tested for anti-proliferative effect of UP-7 using MTS assay. Serum-starved cancer and non-endothelial cells (NCI-H460 human lung cancer, LM-MDA-MB-231 human breast cancer, U87 human brain cancer and HEK293 cells) were treated with UP-7 in a concentration range of 0–400 µM for 30 min, and then stimulated with 5% FBS for proliferation. UP-7 showed no inhibition effects on cancer or non-endothelial cells even at 400 µM similar to UK1, except MDA-MB-231 and LM-MDA-MB-231 (an MDA-MB-231 derivative line) breast cancer cells [18]. Interestingly, UP-7 inhibited specifically the proliferation of MDA-MB-231 and LM-MDA-MB-231 breast cancer cells dose dependently (IC_50_ = 123 µM and IC_50_ = 190 µM, respectively) (Figure 3), although higher concentrations of UP-7 were required for inhibition of proliferation compared to that of HUVECs. These results suggest that UP-7 elicits differential effects on cancer cell proliferation.

### UP-7 inhibits NCI-H460 tumor growth through angiogenesis inhibition

**Figure 4:**
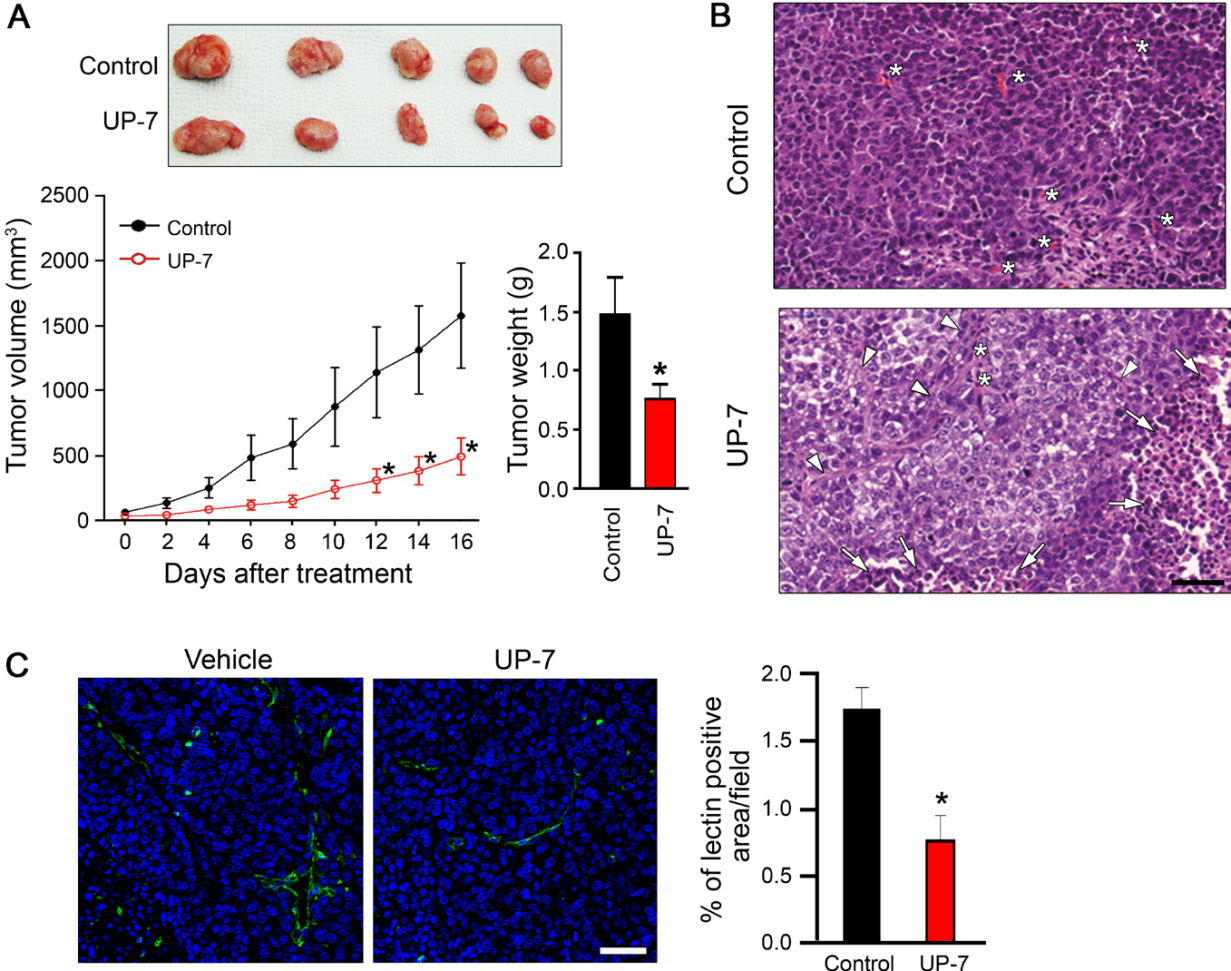
UP-7 inhibits NCI-H460 lung tumor growth *in vivo* NCI-H460 lung cancer cells were injected into 5-week-old mice (5x10^5^ cells per mouse). After solid tumor grew to ∼100 mm^3^, the mice were i.p treated with vehicle alone or UP-7 (50 mg/kg/day). (**A**) Tumor volume was measured every two days (lower left panel). At day 16, tumors were excised from the xenografted mice (upper panel) and weighted (lower right panel). (**B**) H&E staining of the sections from the excised tumors. The representative images are shown. Arrows indicate apoptotic cells, arrow heads fibrous capsule, and asterisks capillaries. Scale bar = 50 μm. (**C**) To detect vessel density, the sections from the excised tumors were stained with FITC-conjugated BS-lectin I (green). Nuclei were stained with DAPI (blue). The representative images are shown in left. Scale bar = 20 μm. Vessel density was calculated using Image J program. The graph represents the relative intensity of BS lectin I-positive area. ^*^p < 0.05 versus control group.

Non-UP-7-sensitive lung cancer cells were chosen for evaluation of the anti-angiogenesis-mediated anti-tumor effect of UP-7 in an *in vivo* xenograft model. After tumors reached a volume of about 100 mm^3^, UP-7 (50 mg/kg) was administered daily for 16 days via intraperitoneal (i.p) injection. UP-7 treated mice showed a strong and significant reduction of tumor volume (69%) and tumor weight (48%) compared to control mice (Figure 4A). To assess if suppression of tumor growth by UP-7 is correlated with its anti-angiogenic activity, tumor sections were stained with hematoxylin and eosin (H&E) or fluorescein isothiocyanate (FITC)-conjugated Griffonia (Bandeiraea) simplicifolia (BS) lectin I. UP-7 treatment produced multiple apoptotic tumor foci composed of pyknotic cells with condensed nuclei (arrows), and the remaining tumor cells were partly differentiated into the features of epithelial cells and divided by encircling with thin fibrous tissue (arrow heads). While the control groups were mostly composed of aggressive tumor cells which were poorly differentiated and synchronically proliferative with abundant capillaries (^*^). Particularly, the UP-7 treated tumor cells showed much reduced mitotic figure compared to the control tumor cells, and they became enlarged into cuboidal to polygonal shape with the increased amount of cytoplasm. The control tumor cells were usually spindle to ovoid in shape with hyperchromatic nuclei, and invasive in the interlacing migratory fashion, whereas the UP-7 treated tumor cells were adhered with each other in the clusters, and showed less hyperchromatic nuclei than the control tumor cells (Figure 4B). BS-lectin I positive blood vessels were also significantly reduced by treatment with UP-7 compared to control (Figure 4C). To quantify the tumor angiogenesis, lectin positive area was analyzed using Image J program. Fluorescence-based quantification showed inhibition of angiogenesis upon UP-7 treatment by about 55% compared to control. These data indicate that UP-7 inhibit NCI-H460 lung tumor growth by inhibiting angiogenesis.

### UP-7 inhibits migration, invasion and FAK activation in breast cancer cells

**Figure 5:**
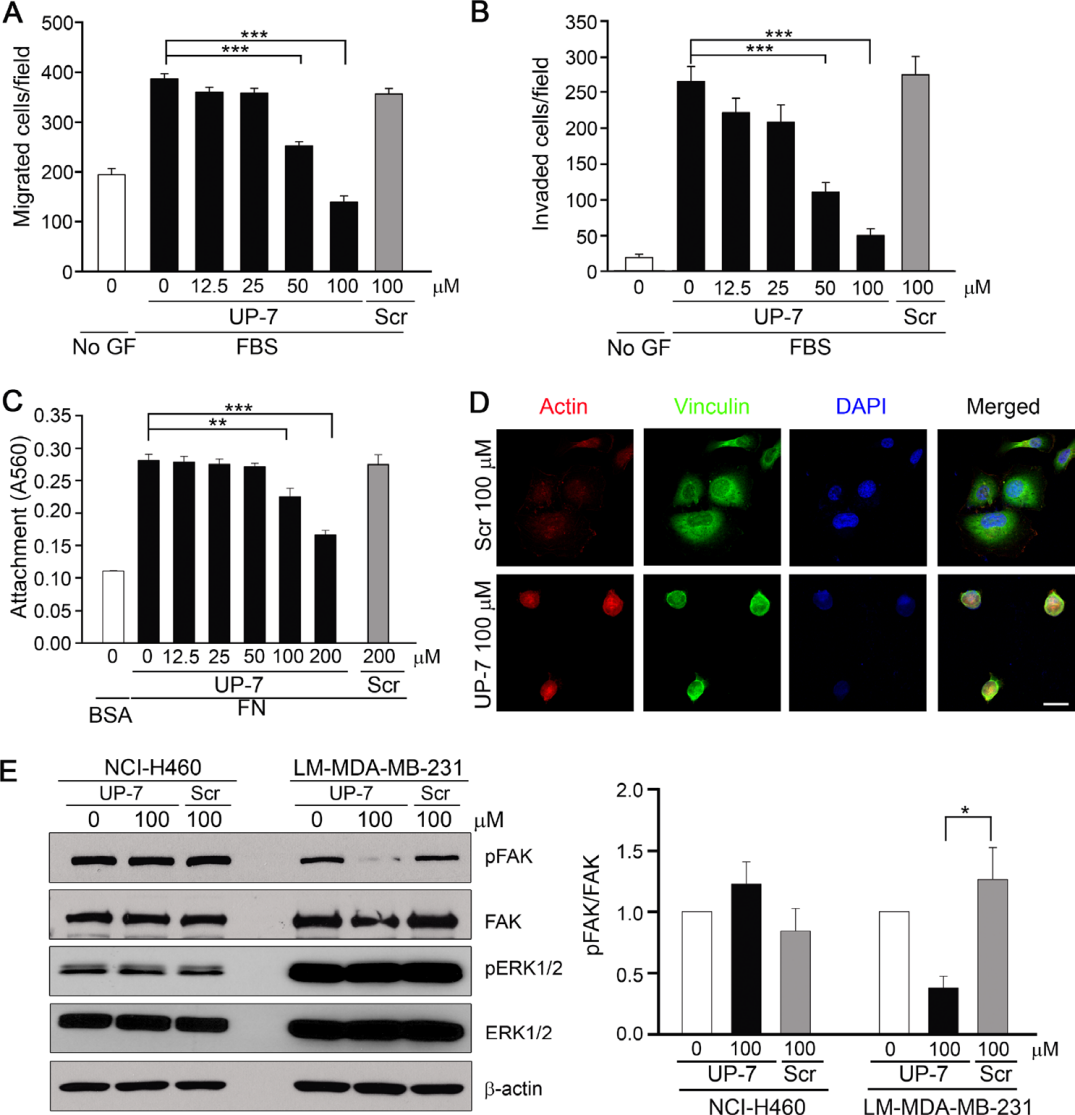
UP-7 inhibits migration, invasion and FAK phosphorylation of LM-MDA-MB-231 breast cancer cells (**A**) LM-MDA-MB-231 cells were pretreated with UP-7 or Scr at various concentrations for 30 min, and then cell migration was induced by 10% FBS for 24 h in transwell. (**B**) LM-MDA-MB-231 cells were pretreated with UP-7 or Scr for 30 min at various concentrations, and then invasion was induced by 10% FBS for 24 h after adding the cells in the Matrigel-loaded transwell insert. (**C**, **D**) Serum-starved LM-MDA-MB-231 cells were pretreated with the indicated concentrations of UP-7 or Scr for 30 min and plated on FN (20 μg/ml)-coated plates for 90 min. The attached cells were stained with crystal violet and incorporated dye was dissolved in 10% acetic acid, followed by measurement of absorbance at 560 nm. Graph represents the absorbance of stained attached cells (C). The attached cells were immunostained with anti-vinculin antibody and Alexa 488 conjugated secondary antibody, followed by staining with TRITC-conjugated phalloidin and DAPI. Scale bar = 20 μm (D). (**E**) Serum-starved NCI-H460 or LM-MDA-MB-231 cells were pretreated with UP-7 or Scr (100 µM) for 30 min and stimulated with 5% FBS for 24 h, followed by western blot analysis. Representative immunoblots (left panel) and quantitative analysis (right panel) of three independent experiments are shown. ^*^p < 0.05; ^**^p < 0.01; and ^***^p < 0.001.

As UP-7 effectively inhibited breast cancer cell proliferation differently from other cancer cells, we investigated the effect of UP-7 on migration and invasion of breast cancer cells. UP-7 significantly inhibited migration and invasion of LM-MDA-MB-231 breast cancer cells in a dose dependent manner (IC_50_ = 41 µM and 42 µM, respectively) (Figure 5A and 5B). Such inhibition was not shown by treatment with Scr. In contrast, UP-7 showed no inhibition effects on migration and invasion of NCI-H460 lung cancer cells (Supplementary Figure 4). As FN plays an essential role in tumor progression and is highly correlated with cancer metastasis [27], we tested the effect of UP-7 on adhesion and spreading of LM-MDA-MB-231 cells onto FN. Interestingly, UP-7 suppressed the adhesion and spreading of LM-MDA-MB-231 cells onto FN (Figure 5C and 5D). Like in HUVECs, UP-7 showed no inhibitory effects on the attachment of LM-MDA-MB231 cells onto immobilized collagen I matrix. We were not able to assess the effect on focal adhesion complex formation of those cells (Supplementary Figure 5), because LM-MDA-MB-231 cells lacked formation of stress fibers on collagen I matrix. In contrast, UP-7 had no impact on attachment and focal adhesion complex formation of NCI-H460 cells on both matrices (Supplementary Figure 6). Since activation of FAK and ERK1/2 was inhibited by UP-7 in HUVECs, we tested whether UP-7 is able to inhibit activation of FAK and ERK1/2 in NCI-H460 and LM-MDA-MB-231 cancer cells. As shown in Figure 5E, UP-7 inhibited phosphorylation of FAK in LM-MDA-MB-231 cells, but not in non-UP-7 sensitive NCI-H460 cells. However, UP-7 showed no effect on ERK1/2 activation since FAK and MEK-ERK signaling events operate independently in MDA-MB-231 cells [28]. Thus, we concluded that UP-7 inhibits migration and invasion of breast cancer cells possibly through inhibition of FAK activation.

### UP-7 suppresses breast cancer cell metastasis to lung tissue.

**Figure 6:**
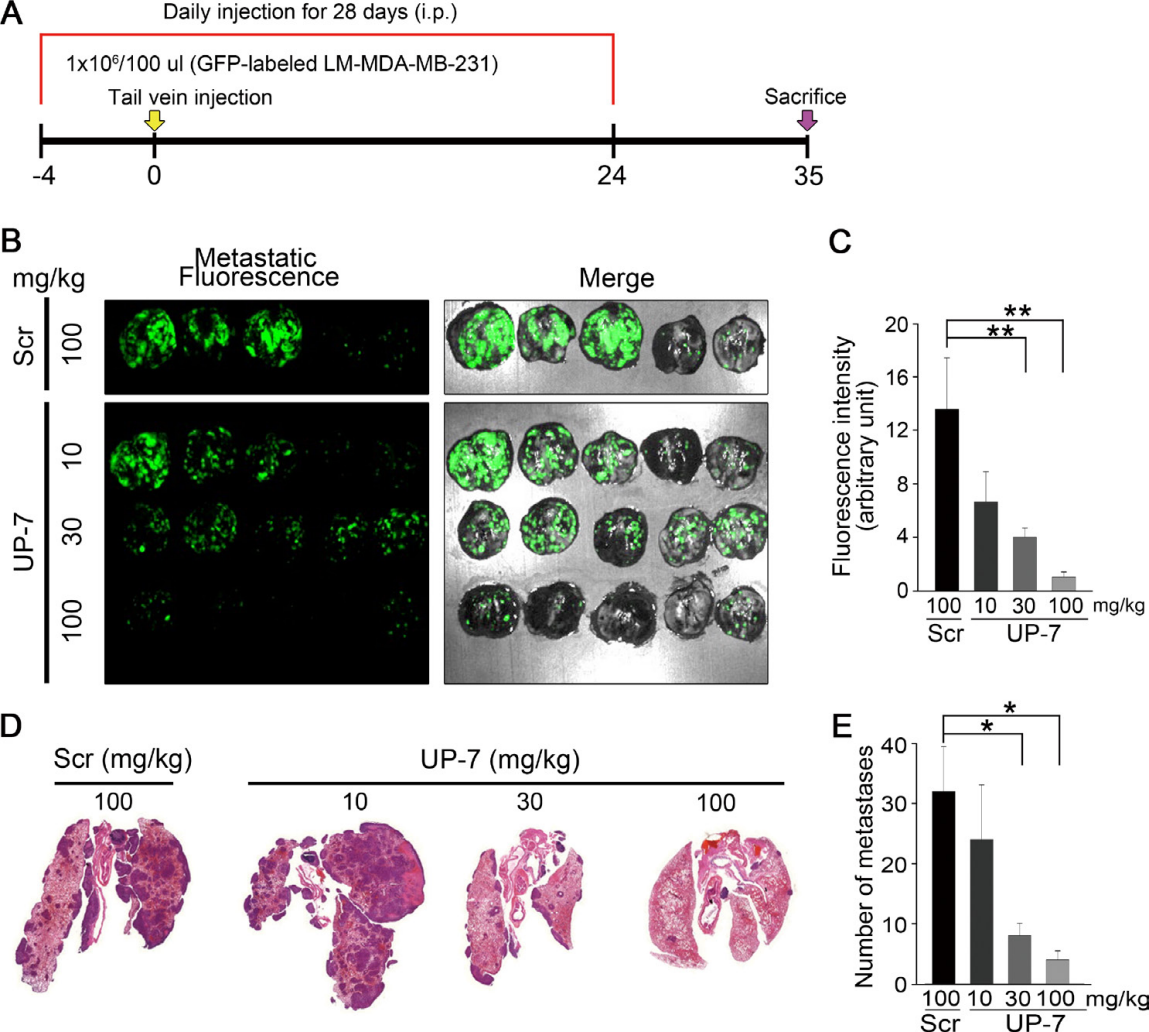
UP-7 inhibits lung metastasis of LM-MDA-MB 231 breast cancer cells (**A**) Schematic of the experimental design. Balb/c nude mice were injected i.p. with UP-7 (10, 30, 100 mg/kg) or Scr (100 mg/kg) by daily for 4 days before implantation, and then LM-MDA-MB-231 cells (1X10^6^) were injected into mice through the tail vein (*n* = 5 mice). Peptide injection continued until day 24. (**B**) Representative fluorescence images of metastatic GFP-labeled LM-MDA-MB-231 tumor-carrying lungs on day 35. Metastatic nodules were visualized as bright GFP-fluorescence. (**C**) Quantification of GFP-positive metastatic nodules using Image J program. (**D**, **E**) Representative images of H&E stained lung tissues from UP-7 or Scr treated mice show metastatic lesions (D) and graph presents the number of lung metastastic lesions (E). ^*^p < 0.05; and ^**^p < 0.01.

Finally, we investigated whether UP-7 may inhibit metastasis of breast cancer cells *in vivo* employing a metastasis animal model. UP-7 (10, 30, 100 mg/kg, i.p) or Scr (100 mg/kg, i.p) was treated daily for 4 days before cell injection and GFP-labeled LM-MDA-MB-231 cells were injected via the tail vein of the nude mice. Daily treatment with UP-7 or Scr was continued for 24 days and the mice were further maintained for 11 days without treatment (Figure 6A). Body weight of the mice treated with UP-7 or Scr did not decrease through the experimental period. The mice were sacrificed and the lungs were excised. Metastatic nodules were observed as white colonies. Bright green fluorescence was co-localized with these metastatic nodules (Supplementary Figure 7; Figure 6B). To compare the lung metastases between UP-7 and Scr treatment groups, fluorescence intensity from the images of lungs were quantitated. UP-7 treated group (100 mg/kg) showed a potent and significant inhibition of fluorescence intensity compared to Scr treated group (1.0 ± 0.3 vs. 13.5 ± 3.8; 92.5% reduction) (Figure 6C). In addition, the number of metastatic lesions (dark purple) in lung tissue was also significantly reduced in the UP-7 treated groups compared with Scr treated group in a dose-dependent manner (Figure 6D and 6E). These data strongly indicate that UP-7 had a drastic inhibitory effect on lung colonization of breast cancer cells.

## DISCUSSION

In the present study, we found that a novel peptide UP-7 derived from extended antiparallel β-sheet structure of UK1 is the most potent anti-angiogenic fragment among UK1 derived peptides. Surprisingly, it inhibited not only primary tumor growth but also metastasis in an experimental breast cancer animal model by blocking of tumor angiogenesis and direct inhibition of growth and invasion of breast cancer cells. Such data strongly suggest that UP-7 can be used as an effective anti-angiogenic agent and more importantly that it can be developed as a valuable anti-metastatic agent against breast cancer.

It is notable that UP-7 which has the most potent anti-angiogenic activity is derived from the extended antiparallel β-sheet structure of UK1, because the most potent anti-angiogenic activity has been also shown in the antiparallel β-sheet structure-derived peptide (TP-7) among the peptides derived from the tissue type plasminogen activator kringle 2 domain [22]. The 33 mer peptide anginex that has been designed by using basic folding principles and incorporating short sequences from the β-sheet domains of anti-angiogenic proteins also demonstrates a potent anti-angiogenic activity [29]. It shows a potent antitumor activity in the syngeneic mouse B16F10 melanoma model and in a xenograft human tumor model [30]. Furthermore, several anti-angiogenic proteins such as endostatin, platelet factor 4 and tumor necrosis factor, are comprised primarily of anti-parallel β-sheet structure [31]. The functional key residues of anti-angiogenic anginex are hydrophobic residues i.e. Leu5, Val7, Ile20, Val22 and Leu24 [32]. Interestingly, UP-7 derived from β-sheet motif of UK1 has 7 hydrophobic residues (PWCYVQVGLKPLVQE, Pro1, Val5, Val7, Leu9, Pro11, Leu12 and Val13), which may provide key residues responsible for its anti-angiogenic activity.

Notably, UP-7 markedly inhibited cellular adhesion onto FN-coated plate and FN-induced cytoskeleton rearrangement in both HUVECs and LM-MDA-MB-231 breast cancer cells. FN expression is highly correlated with tumorigenesis and metastasis, and interaction of cancer cells with FN is required for metastasis [33]. Various peptides that mimic the cell adhesive region of FN have been developed to inhibit metastasis [34–36]. FN is recognized by at least ten integrin family including integrin αvβ3 and α5β1 [37]. A small cyclic pentapeptide, cilengitide, RGD-containing peptide that selectively inhibits αvβ3 and αvβ5 integrins, has been shown to inhibit VEGF- and bFGF-induced migration and tube formation of ECs [38, 39]. RGD peptide reduces bFGF-dependent chemotaxis [∼65% inhibition at 250 μg/ml (∼577 µM)] and proliferation [50% inhibition at 500 μg/ml (∼1,154 µM)] at high concentrations [40] compared to UP-7, which inhibits migration and proliferation of HUVECs with the IC_50_ of 15∼16 µM and 25∼40 µM, respectively. In the previous study, we have shown that UK1, mother molecule of UP-7, interacts with integrin αvβ3 but not with α5β1 in ECs. However, its anti-migratory activity on ECs is not related with integrin αvβ3 [41]. Since pretreatment of UP-7 had no effect on adhesion of HUVECs on immobilized UK1, there is a possibility that UP-7 may act on ECs differently from UK1 (data not shown). UP-7 also showed no inhibition of *in vitro* tube formation of HUVECs on Matrigel albeit its potent *in vivo* anti-angiogenic activity, implying that UP-7 acts on ECs differently from cilengitide or TP-7. It is also notable that UP-7 also more potently inhibits proliferation, migration and invasion of LM-MDA-MB-231 breast tumor cells with an IC_50_ of 190 µM, 41 µM and 42 µM respectively: cilengitide suppresses proliferation (at 340 µM, 65.5% inhibition), migration (at 340 µM, 26.9% inhibition) and invasion (at 340 µM, 17.2% inhibition) of MDA-MB-231 breast cancer cells at higher concentrations [42]. Interestingly, UP-7 also disturbed the formation of focal adhesion complex on collagen I matrix in endothelial cells. Such data support that UP-7 is a novel potent anti-angiogenic, anti-tumoric, and anti-metastatic peptide that undergoes different mechanism of action from cilengitide.

FAK expression and activity in ECs is important during vascular development and tumor angiogenesis. mRNA level of FAK is also increased in invasive breast cancers [6]. Since FAK is at the intersection of various signaling pathways that promote cancer growth and metastasis, FAK is considered to be a therapeutic target in cancers. In this study, we have shown that UP-7 inhibits phosphorylation of FAK regardless of growth factor types in both ECs and LM-MDA-MB-231 breast cancer cells, which may be linked to its anti-angiogenic and anti-tumoric activity. In the future, further studies are needed to elucidate the mechanism by which UP-7 inhibits activation of FAK.

In the *in vivo* xenograft tumor model of human lung cancer NCI-H460 cells, UP-7 showed inhibition of *in vivo* tumor growth, although it did not affect directly cancer cell growth *in vitro*. We presume that it occurs probably by inhibition of angiogenesis because UP-7 treatment resulted in reduced vessel density compared to control group. Because the experimental metastasis model used in this study addresses the event post intravasation stage [43], anti-metastatic activity of UP-7 may be due to its multiple inhibitory effects on cellular adhesion onto extracellular matrix, migration and invasion, and angiogenic activity required for the settlement and growth of LM-MDA-MB-231 breast cancers at the target site. It is needed to study how differential effects of UP-7 exert on cancer cells, with precise mechanism of action of UP-7.

In our study, we showed that UP-7 is a strong candidate for metastatic breast cancer therapy owing to its multiplicity of activities. UP-7 inhibited proliferation, migration, and adhesion onto FN and also inhibited phosphorylation of FAK both in endothelial cell and breast cancer cells. Cyclization of peptides confers increase of resistance to proteolytic degradation and an improved bioavailability [44]. As there is possibility that the cyclic conformation of the UP-7 peptide may confer anti-angiogenic or anti-tumor activities at lower concentrations by increase of stability, further study will be investigated on this issue. Anti-parallel β-sheet structure with hydrophobic residues may be key elements for anti-angiogenic and anti-tumoric activities of UP-7 that may undergo different mechanism of action from cilengitide. Further studies are needed to determine which molecules are involved in the direct interaction of UP-7 with endothelial cells or breast cancer cells. Taken together, we strongly suggest that a novel peptide UP-7 can be used as an effective therapeutic agent for treatment of breast cancer metastasis.

## MATERIALS AND METHODS

### Peptide synthesis

Peptides were designed based on the structure of UK1 and characteristics of amino acid residues in the kringle domain of urokinase [21]. The peptides were synthesized by Peptron (Daejeon, Korea), dissolved in distilled water or dimethyl sulfoxide (DMSO), and stored at −70 C until use.

### Cell culture

HUVECs were isolated from human cords according to a procedure described previously [45]. HUVECs were cultured in M199 medium supplemented with 20% fetal bovine serum (FBS, Gibco, Grand Island, NY), 30 μg/ml endothelial cell growth supplements (BD Biosciences, Franklin Lakes, NJ), and 90 μg/ml heparin (Sigma, St. Louis, MO). MDA-MB-231 (# 30026) and NCI-H460 (# 30177) cells were purchased from Korean Cell Line Bank (Seoul, South Korea), and U87 (# HTB-14) and HEK293 (# CRL-1573) cells from ATCC (Manassas, VA). LM-MDA-MB-231 cells were originated from MDA-MB-231 cells metastasized to mouse lungs and transduced with green fluorescence protein (GFP). Cancer cell lines were cultured in DMEM supplemented with 10% FBS.

### Reagents and antibodies

Polyclonal antibodies to phospho-FAK (Y397) and FAK were obtained from R&D (Minneapolis, MN) and Millipore (Billerica, MA), respectively, polyclonal antibodies to phospho-ERK1/2 and ERK1/2 from Cell Signaling Technology (Beverly, MA), monoclonal antibody to β-actin from Sigma (St. Louis, MO) and polyclonal anti-mouse HRP and anti-rabbit HRP from Santa Cruz Biotechnology (Santa Cruz, CA). Actin cytoskeleton and focal adhesion staining kit (FAK100) was purchased from Millipore and polyclonal anti-mouse Alexa Fluor 488 from Invitrogen (Carlsbad, CA). Human recombinant vascular endothelial growth factor (VEGF) and basic fibroblast growth factor were purchased from Peprotech (Rocky Hill, NJ), FN from BD Biosciences, and collagen I from Gibco (Grand Island, NY).

### Cell proliferation assay

1×10^3^ cells were plated in each well of a 96-well plate. After 24 ∼48 h, the cells were serum starved for 4 h, and then treated with indicated peptides for 30 min. Then, the cells were treated with 3 ng/ml bFGF or 5 ng/ml VEGF and 5% FBS (HUVECs), or 5% FBS (cancer cells) for 72 h. Proliferation of the cells was assessed using the MTS Cell Proliferation Assay Kit (Promega Corp., Madison, WI).

### Migration and invasion assays

Cell migration assay was performed using a modified Boyden chamber (48 well) or Transwell (Corning Costar, Cambridge, MA) as described previously [46]. Serum-starved cells were incubated with each peptide for 30 min, placed in the upper chamber and allowed to migrate toward VEGF (5 ng/ml) or bFGF (10 ng/ml) (HUVECs) or toward 10% FBS (other cancer cells). After 5 ∼ 8 h (HUVECs) or 24 h (cancer cells), the migrated cells were fixed and stained with Diff-Quik solution (Sysmex Co., Kobe, Japan). The migrated cells were photographed and counted in five randomly selected fields. For invasion assay, we used the same procedure except coating the upper side of the microporous membrane of Transwell insert (8 μm) with 100 μl or the top chamber of 48 well microchemotaxis chamber with 20 μl of the diluted Matrigel (1:2, Matrigel: DMEM). Briefly, the inner surface of Transwell or the top chamber of 48 well microchemotaxis chamber was coated with Matrigel diluted with serum free DMEM media (1:2) for 30 min. UP-7 or Scr peptide treated LM-MDA-MB-231 or NCI-H460 cells were added onto Matrigel coated inner surface and invasion of the cells was induced by 10% FBS for 24 h. The invaded cells were fixed and stained with Diff-Quik solution. The invaded cells were photographed and counted in five randomly selected fields.

### In vivo Matrigel plug assay

All procedures were performed in accordance with the policies of the Catholic University of Korea institutional animal care and use committee. This assay was performed as described previously [22]. Seven-week-old C57BL/6 mice (SLC, Japan) were injected subcutaneously in the midventral abdominal region with 0.6 ml of Matrigel (BD Bioscience) containing bFGF (100 ng/ml), VEGF (100 ng/ml), and heparin (18 units) with or without 100 µM of UP-7 (*n* = 8 per group). After 7 days, the Matrigel plugs were removed and photographed. To evaluate formation of the vessels, the amount of hemoglobin was quantified using Drabkin’s reagent (Sigma).

### Western blot analysis

Serum-starved HUVECs were incubated with UP-7 or Scr for 30 min and then treated with bFGF (3 ng/ml) or VEGF (5 ng/ml) for 10 min. Serum starved cancer cells were incubated with UP-7 or Scr and then incubated with 5% FBS for 24 h. Western blotting assay was performed as described previously [46].

### Immunofluorescence assay

HUVECs (2x10^4^) were seeded on gelatin-coated glass cover slips in the EGM-2 medium. After 24 h, the cells were starved in the serum-free EBM-2 medium for 4 h and then treated with UP-7 or Scr for 30 min. After that, the cells were incubated with 3 ng/ml bFGF or 5 ng/ml VEGF for 10 min. After washes with PBS, the cells were fixed and were stained with Actin cytoskeleton and focal adhesion staining kit (FAK100, Millipore) according to the manufacturer’s instructions. The fluorescent images were obtained using confocal microscopy (Zeiss LSM 510 Meta with LSM image examiner software, Germany) at × 800 magnification.

### Cell adhesion assay

Cell adhesion assay was performed as described previously [46]. 96-well or 24 well-plates were coated with FN (20 μg/ml) (BD Bioscience) or collagen type I (50 μg/ml) (Gibco BRL) for 16–18 h at 4°C. Detached cells (1 × 10^4^) were pretreated with either UP-7 or Scr and plated on the coated plates, followed by subsequent incubation for 90 min. After washes with PBS, the attached cells were fixed and stained with crystal violet. The incorporated dye was dissolved in 10% acetic acid followed by measurement of absorbance at 560 nm or stained cells were counted. For staining of the actin cytoskeleton, UP-7 or Scr peptide pretreated detached cells (3 × 10^4^) were seeded on coverslips coated with either FN (20 μg/mL) or collagen I (50 μg/mL) in 24-well plates, followed by subsequent incubation for 90 min. The attached cells were fixed and were stained with Actin cytoskeleton and focal adhesion staining kit (FAK100, Millipore) according to the manufacturer’s instructions.

### Xenograft tumor model

All procedures were performed in accordance with the policies of the Catholic University of Korea Institutional Animal Care and Use Committees. 5-week-old female BALB/c nude mice (SLC) were injected with NCI-H460 cells (5 × 10^5^cells per mouse) into the right flank subcutaneously. Tumor volume was quantified every two days with electronic caliper. After solid tumor grew to ∼100 mm^3^, treatments were performed by intraperitoneal (i.p.) injection with vehicle alone or UP-7 peptide (50 mg/kg) every day for 16 days. Tumor size was calculated as width^2^ × length × 0.52 (mm^3^).

### Histological analysis

Tumor paraffin sections (5 μm) were rehydrated and were subjected to antigen retrieval by heating in citration solution (pH 6.0). The sections were blocked by 5% normal goat serum and incubated with FITC-conjugated BS lectin I (Vector Laboratories, Brulingame, CA) for 1 h at room temperature. The slides were counterstained with 1 μg/ml DAPI (Sigma). For analysis of nucleus and cytoplasm in tumor tissue, sections were stained with H&E.

### Experimental metastasis model

All procedures were performed in accordance with the policies of the Catholic University of Korea institutional animal care and use committee. UP-7 or Scr peptide was pretreated into BALB/c nude mice (SLC) daily by i.p injection for 4 days. GFP-labeled LM-MDA-MB-231 cells (1 × 10^6^) were suspended into 100 μl of saline and injected intravenously into the tail vein of the mice. Then, peptide was injected i.p. every day for 24 days and the mice were sacrificed 11 days after last injection. Lungs were excised and the fluorescence signal intensity was measured with an *in vivo* imaging system (Maestro, CRi Inc., Woburn, Ma). Intensity of fluorescence from metastatic burden was quantitated by using Image J program.

### Statistical analysis

The data were presented as mean ± SEM. Student’s *t* test was used to compare all pairwise samples. For multiple comparisons, one-way ANOVA was performed. ^*^ *p* < 0.05, ^**^p < 0.01, or ^***^p < 0.001 was assumed to denote statistical significance.

## SUPPLEMENTARY MATERIALS FIGURES AND TABLE



## References

[1] Scully OJ, Bay BH, Yip G, Yu Y (2012). Breast cancer metastasis. Cancer Genomics Proteomics.

[2] Akiyama SK, Olden K, Yamada KM (1995). Fibronectin and integrins in invasion and metastasis. Can Metastasis Rev.

[3] van Roosmalen W, Le Devedec SE, Golani O, Smid M, Pulyakhina I, Timmermans AM, Look MP, Zi D, Pont C, de Graauw M, Naffar-Abu-Amara S, Kirsanova C, Rustici G (2015). Tumor cell migration screen identifies SRPK1 as breast cancer metastasis determinant. J Clin Invest.

[4] van Zijl F, Krupitza G, Mikulits W (2011). Initial steps of metastasis: cell invasion and endothelial transmigration. Mutat Res.

[5] Eliceiri BP (2001). Integrin and growth factor receptor crosstalk. Cir Res.

[6] Sulzmaier FJ, Jean C, Schlaepfer DD (2014). FAK in cancer: mechanistic findings and clinical applications. Nat Rev Cancer.

[7] Tavora B, Batista S, Reynolds LE, Jadeja S, Robinson S, Kostourou V, Hart I, Fruttiger M, Parsons M, Hodivala-Dilke KM (2010). Endothelial FAK is required for tumour angiogenesis. EMBO Mol Med.

[8] Ilic D, Furuta Y, Kanazawa S, Takeda N, Sobue K, Nakatsuji N, Nomura S, Fujimoto J, Okada M, Yamamoto T (1995). Reduced cell motility and enhanced focal adhesion contact formation in cells from FAK-deficient mice. Nature.

[9] Cary LA, Chang JF, Guan JL (1996). Stimulation of cell migration by overexpression of focal adhesion kinase and its association with Src and Fyn. J Cell Sci.

[10] Kaplan RN, Riba RD, Zacharoulis S, Bramley AH, Vincent L, Costa C, MacDonald DD, Jin DK, Shido K, Kerns SA, Zhu Z, Hicklin D, Wu Y (2005). VEGFR1-positive haematopoietic bone marrow progenitors initiate the pre-metastatic niche. Nature.

[11] Shibuya M (2014). VEGF-VEGFR Signals in Health and Disease. Biomol Ther (Seoul).

[12] Senger DR, Davis GE (2011). Angiogenesis. Cold Spring Harb Perspct Biol.

[13] Brooks PC, Clark RA, Cheresh DA (1994). Requirement of vascular integrin alpha v beta 3 for angiogenesis. Science.

[14] Desgrosellier JS, Cheresh DA (2010). Integrins in cancer: biological implications and therapeutic opportunities. Nat Rev Cancer.

[15] Reynolds AR, Hart IR, Watson AR, Welti JC, Silva RG, Robinson SD, Da Violante G, Gourlaouen M, Salih M, Jones MC, Jones DT, Saunders G, Kostourou V (2009). Stimulation of tumor growth and angiogenesis by low concentrations of RGD-mimetic integrin inhibitors. Nat Med.

[16] Chinot OL (2014). Cilengitide in glioblastoma: when did it fail?. Lancet Oncol.

[17] Ferrara N (2010). Pathways mediating VEGF-independent tumor angiogenesis. Cytokine Growth Factor Rev.

[18] Kim KS, Hong YK, Joe YA, Lee Y, Shin JY, Park HE, Lee IH, Lee SY, Kang DK, Chang SI, Chung SI (2003). Anti-angiogenic activity of the recombinant kringle domain of urokinase and its specific entry into endothelial cells. J Biol Chem.

[19] Kim CK, Hong SH, Joe YA, Shim BS, Lee SK, Hong YK (2007). The recombinant kringle domain of urokinase plasminogen activator inhibits *in vivo* malignant glioma growth. Cancer Sci.

[20] Fairlie DP, West ML, Wong AK (1998). Towards protein surface mimetics. Curr Med Chem.

[21] Li X, Bokman AM, Llinas M, Smith RA, Dobson CM (1994). Solution structure of the kringle domain from urokinase-type plasminogen activator. J Mol Biol.

[22] Kim HK, Choi JS, Lee SW, Joo CK, Joe YA (2017). A Novel Peptide Derived From Tissue-Type Plasminogen Activator Potently Inhibits Angiogenesis and Corneal Neovascularization. J Cell Biochem.

[23] Kim CK, Joe YA, Lee SK, Kim EK, O E, Kim HK, Oh BJ, Hong SH, Hong YK (2010). Enhancement of anti-tumor activity by low-dose combination of the recombinant urokinase kringle domain and celecoxib in a glioma model. Cancer Lett.

[24] Kim BM, Lee DH, Choi HJ, Lee KH, Kang SJ, Joe YA, Hong YK, Hong SH (2012). The recombinant kringle domain of urokinase plasminogen activator inhibits VEGF165-induced angiogenesis of HUVECs by suppressing VEGFR2 dimerization and subsequent signal transduction. IUBMB Life.

[25] Bischoff J (1997). Cell adhesion and angiogenesis. J Clin Invest.

[26] Lamalice L, Le Boeuf F, Huot J (2007). Endothelial cell migration during angiogenesis. Cir Res.

[27] Wu CI, Chang MM, Su CL, Ling P, Chang WT, Cheng HC (2014). Impacts of protease inhibitors on clathrin and fibronectin in cancer metastasis. Biomarkers and Genomic Medicine.

[28] Bianchi-Smiraglia A, Paesante S, Bakin AV (2013). Integrin beta5 contributes to the tumorigenic potential of breast cancer cells through the Src-FAK, MEK-ERK signaling pathways. Oncogene.

[29] Griffioen AW, van der Schaft DW, Barendsz-Janson AF, Cox A, Struijker Boudier HA, Hillen HF, Mayo KH (2001). Anginex, a designed peptide that inhibits angiogenesis. Biochem J.

[30] van der Schaft DW, Dings RP, de Lussanet QG, van Eijk LI, Nap AW, Beets-Tan RG, Bouma-Ter Steege JC, Wagstaff J, Mayo KH, Griffioen AW (2002). The designer anti-angiogenic peptide anginex targets tumor endothelial cells and inhibits tumor growth in animal models. FASEB J.

[31] Dings RP, Arroyo MM, Lockwood NA, van Eijk LI, Haseman JR, Griffioen AW, Mayo KH (2003). Beta-sheet is the bioactive conformation of the anti-angiogenic anginex peptide. Biochem J.

[32] Mayo KH, van der Schaft DW, Griffioen AW (2001). Designed beta-sheet peptides that inhibit proliferation and induce apoptosis in endothelial cells. Angiogenesis.

[33] Clark EA, Golub TR, Lander ES, Hynes RO (2000). Genomic analysis of metastasis reveals an essential role for RhoC. Nature.

[34] Humphries MJ, Olden K, Yamada KM (1986). A synthetic peptide from fibronectin inhibits experimental metastasis of murine melanoma cells. Science.

[35] Tang NH, Chen YL, Wang XQ, Li XJ, Wu Y, Zou QL, Chen YZ (2010). N-terminal and C-terminal heparin-binding domain polypeptides derived from fibronectin reduce adhesion and invasion of liver cancer cells. BMC cancer.

[36] Saiki I, Murata J, Makabe T, Matsumoto Y, Ohdate Y, Kawase Y, Taguchi Y, Shimojo T, Kimizuka F, Kato I, Azuma I (1990). Inhibition of lung metastasis by synthetic and recombinant fragments of human fibronectin with functional domains. Jap J Cancer Res.

[37] Johansson S, Svineng G, Wennerberg K, Armulik A, Lohikangas L (1997). Fibronectin-integrin interactions. Front Biosci.

[38] Dechantsreiter MA, Planker E, Matha B, Lohof E, Holzemann G, Jonczyk A, Goodman SL, Kessler H (1999). N-Methylated cyclic RGD peptides as highly active and selective alpha(V)beta integrin antagonists. J Med Chem.

[39] Nisato RE, Tille JC, Jonczyk A, Goodman SL, Pepper MS (2003). alphav beta 3 and alphav beta 5 integrin antagonists inhibit angiogenesis *in vitro*. Angiogenesis.

[40] Aguzzi MS, Giampietri C, De Marchis F, Padula F, Gaeta R, Ragone G, Capogrossi MC, Facchiano A (2004). RGDS peptide induces caspase 8 and caspase 9 activation in human endothelial cells. Blood.

[41] O E, Kim HK, Hong SH, Kim CK, Hong YK, Joe YA (2008). Integrin alphavbeta3 is not significantly implicated in the anti-migratory effect of anti-angiogenic urokinase kringle domain. Oncol Rep.

[42] Bretschi M, Merz M, Komljenovic D, Berger MR, Semmler W, Bauerle T (2011). Cilengitide inhibits metastatic bone colonization in a nude rat model. Oncol Rep.

[43] Zibara K, Awada Z, Dib L, El-Saghir J, Al-Ghadban S, Ibrik A, El-Zein N, El-Sabban M (2015). Anti-angiogenesis therapy and gap junction inhibition reduce MDA-MB-231 breast cancer cell invasion and metastasis *in vitro* and *in vivo*. Sci Rep.

[44] Buerkle MA, Pahernik SA, Sutter A, Jonczyk A, Messmer K, Dellian M (2002). Inhibition of the alpha-nu integrins with a cyclic RGD peptide impairs angiogenesis, growth and metastasis of solid tumours *in vivo*. Br J Cancer.

[45] Jaffe EA, Nachman RL, Becker CG, Minick CR (1973). Culture of human endothelial cells derived from umbilical veins. Identification by morphologic and immunologic criteria. J Clin Invest.

[46] Kim HK, Oh DS, Lee SB, Ha JM, Joe YA (2008). Antimigratory effect of TK1–2 is mediated in part by interfering with integrin alpha2beta1. Mol Cancer Ther.

