# Supplementary material for: Urokinase-derived peptide UP-7 suppresses tumor angiogenesis and metastasis through inhibition of FAK activation
Source: Oncotarget. 2018 Jan 10;9(11):9951–62. doi: 10.18632/oncotarget.24131 (PMC5839413; doi:10.18632/oncotarget.24131)
Supplement: Supplementary file 1 [file oncotarget-09-9951-s001.pdf]

Urokinase-derived peptide UP-7 suppresses tumor angiogenesis and metastasis through inhibition of FAK activation

SUPPLEMENTARY MATERIALS

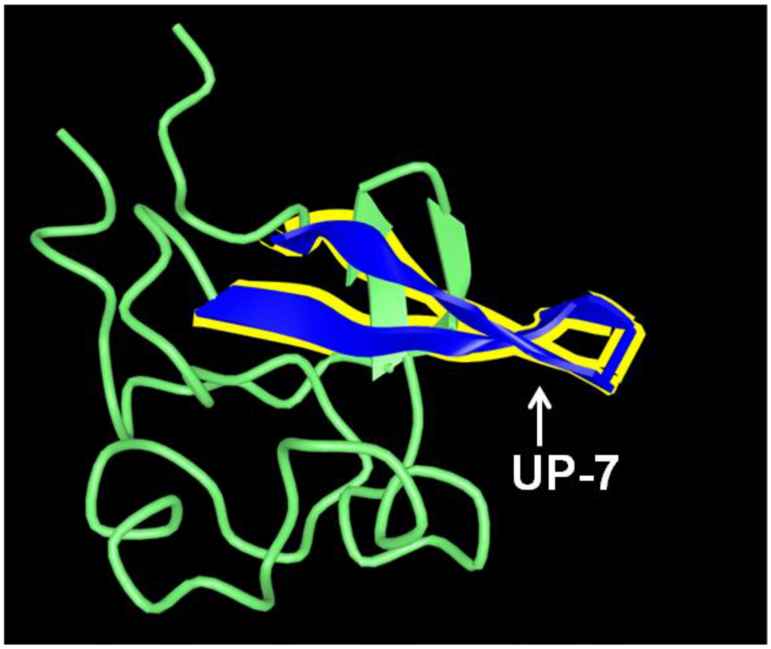

Supplementary Figure 1: 3 D structure of UK1 and location of UP-7 peptide sequence. The 3D structure of UK1 prepared from the NCBI's molecular modeling database using a Cn3D software, NCBI-developed 3D molecular visualization program. An arrow indicates the UP-7 peptide sequence with β -sheet structure.

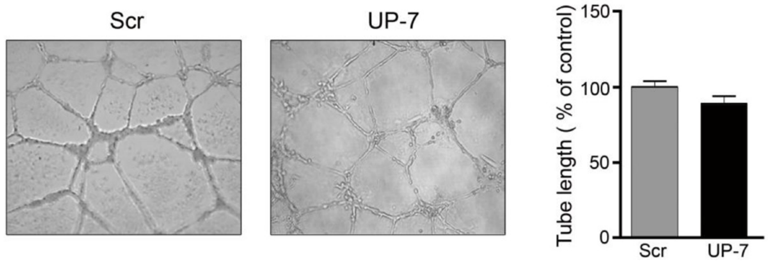

Supplementary Figure 2: No inhibitory effect of UP-7 on tube formation of HUVECs. Serum-starved HUVECs were detached and incubated with UP-7 or Scr (100 μM) for 30 min. Then, the cells were seeded on a Matrigel-coated plate, and incubated for 12 h. The representative fields (left) are shown, and graph presents relative percentage of total tube length compared with Scr peptide treatment (right)

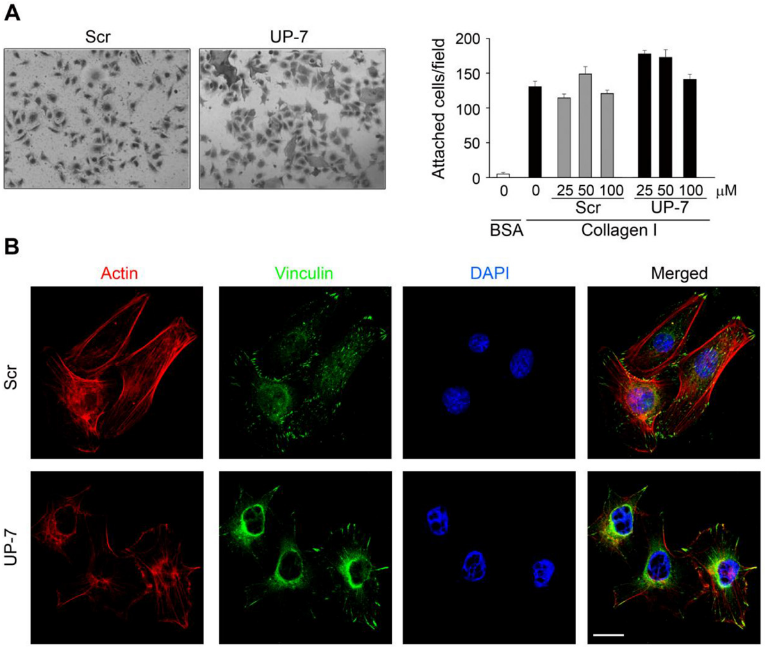

Supplementary Figure 3: The effect of UP-7 on attachment and focal adhesion formation of HUVECs on collagen I matrix. Serum-starved HUVECs were pretreated with the indicated concentrations of UP-7 or Scr for 30 min and seeded on collagen I (50 μg/ml)-coated plates for 90 min. (**A**) After washing, the attached cells were stained with crystal violet and counted. Representative images (left panel) and the number of attached cells (right panel) are shown. (**B**) The attached cells were immunostained with anti-vinculin antibody and Alexa 488-conjugated secondary antibody, followed by staining with TRITC-conjugated phalloidin and DAPI. Scale bar = 20 μm.

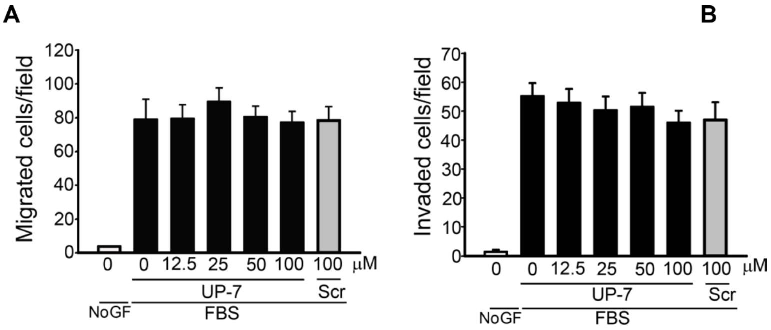

Supplementary Figure 4: No inhibitory effect of UP-7 on migration and invasion of NCI-H460 lung cancer cells. NCI-H460 cells were treated with the indicated concentrations of UP-7 or Scr, and migration (A) or invasion (B) was induced by 10% FBS for 24 h.

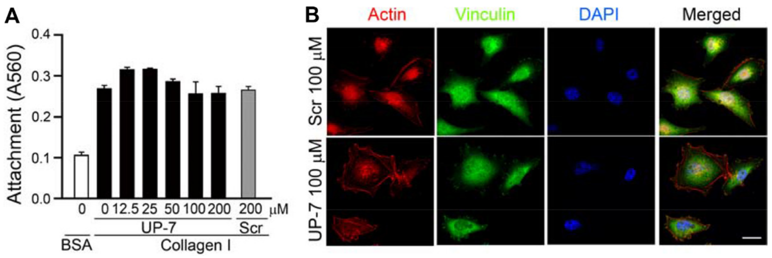

Supplementary Figure 5: The effect of UP-7 on attachment and focal adhesion formation of LM-MDA-MB-231 breast cancer cells on collagen I matrix. Serum-starved LM-MDA-MB-231 cells were pretreated with the indicated concentrations of UP-7 or Scr for 30 min and plated on collagen I (50 μg/m)-coated plates for 90 min. (A) The attached cells were stained with crystal violet and incorporated dye was dissolved in 10% acetic acid, followed by measurement of absorbance at 560 nm. (B) The attached cells were immunostained with anti-vinculin antibody and Alexa 488-conjugated secondary antibody, followed by staining with TRITC-conjugated phalloidin and DAPI. Scale bar = 20 μm.

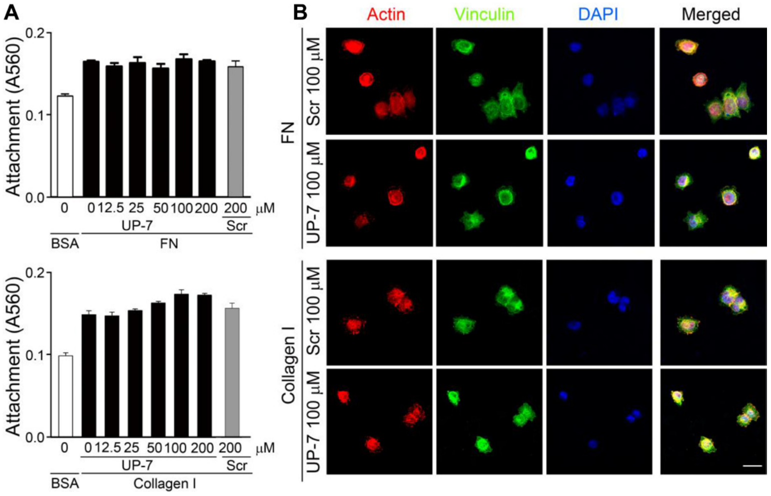

Supplementary Figure 6: No inhibitory effect of UP-7 on attachment and spreading of NCI-H460 lung cancer cells on FN and collagen I matrices. Serum-starved NCI-H460 cells were pretreated with UP-7 or Scr for 30 min and plated on FN (20 mg/ml) or collagen I (50 μg/ml)-coated plates for 90 min. (**A**) The attached cells were stained with crystal violet and incorporated dye was dissolved in 10% acetic acid, followed by measurement of absorbance at 560 nm. (**B**) The attached cells were immunostained with anti-vinculin antibody and Alexa 488-conjugated secondary antibody, followed by staining with TRITC-conjugated phalloidin and DAPI. Scale bar = 20 μm.

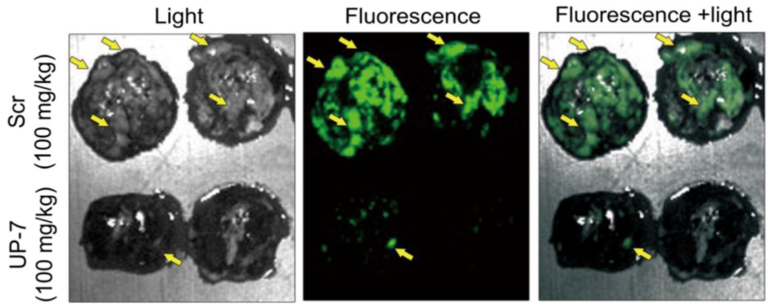

Supplementary Figure 7: Visualization of lung metastatic nodules of GFP-labeled LM-MDA-MB-231 breast cancer cells White colonies of lung metastases of GFP-labeled LM-MDA-MB-231 breast cancer cells were colocalized with green fluorescence images.

Supplementary Table 1: Amino acid sequences of UK1-derived peptides and their anti-endothelial activity (mean ± SEM)

Peptide (100 µM)	Sequences	Proliferation (%)[a]	Migration (%)[b]
UP-1	EGNGHFY	100.6 ± 5.4	114.0 ± 11.1
UP-2	STDTMGRP	99.9 ± 1.6	101.4 ± 10.4
UP-3	PWNSATVLQQTYHAH	74.0 ± 7.6	64.3 ± 1.3
UP-4	SDALQLG	109.4 ± 6.3	130.3 ± 13.8
UP-5	GKHNY	83.0 ± 5.6	69.7 ± 4.8
UP-6	PDNRRR	89.4 ± 4.0	72.4 ± 9.4
UP-7	PWCYVQVGLKPLVQE	7.6 ± 5.6	7.7 ± 0.9

Displayed results indicate relative percentage compared with untreated control. [a]For proliferation assay of HUVECs, 10 ng/ml of bFGF was used as stimulator. Cell proliferation was measured by MTS assay as described in Materials and Methods. [b]For migration assay, HUVECs were pretreated with each peptide and placed on the upper chamber. 5 ng/ml of VEGF was used to induce cell migration for 5 h in 48 well chemotaxis Boyden chamber. The migrated cells were counted.
